# Predicting the Intention to Pursue Certified Professional Accountancy Qualification Among the Accounting Students

**DOI:** 10.3389/fpsyg.2022.860204

**Published:** 2022-03-15

**Authors:** Tiw Kai Chi, Thai Sin Yi, Abdullah Al Mamun, Naeem Hayat, Anas A. Salamah, Qing Yang

**Affiliations:** ^1^Faculty of Business and Management, UCSI University, Kuala Lumpur, Malaysia; ^2^UKM – Graduate School of Business, Universiti Kebangsaan Malaysia, Bangi, Malaysia; ^3^Faculty of Entrepreneurship and Business, Universiti Malaysia Kelantan, Kota Bharu, Malaysia; ^4^College of Business Administration, Prince Sattam Bin Abdulaziz University, Al-Kharj, Saudi Arabia; ^5^UCSI Graduate Business School, UCSI University, Cheras, Malaysia

**Keywords:** accounting, certified professional accountancy qualification, capabilities, career opportunities, accounting student

## Abstract

The global progress empowers the development of new business and expansion of existing business. The availability of sufficient accounting professional are necessary to manage and document the business activities. However, youth are less inclined to purse accounting as profession to keep the progress of global and local economic development. The current study aimed to explore the formation of the intention to pursue Certified Professional Accountancy Qualification (CPAQ) with factor of capabilities, career opportunities, job security with respect to the extended theory of planned behavior (TPB), i.e., attitude, subjective norms and perceived behavioral control. The study adopted a cross-sectional design and collected quantitative data from a total of 339 accounting students from Malaysia using an online survey. The finding revealed that capabilities and career opportunities influenced the students’ intention to pursue CPAQ. Meanwhile, perceived behavioral control had significantly affect the students’ decision to pursue CPAQ, which is in line with the TPB. The study concentrated on the importance of these factors in influencing the students’ intention and decision to pursue CPAQ. The study offered vital implications for accounting educators and educational institutions to promote the accounting profession as choice and students engage in pursuing CPAQ. The Malaysian government should encourage and support accounting students financially for pursuing CPAQ by providing job security and enhancing subjective norms that may enable these students to complete the required professional qualifications. The study’s limitations and future research opportunities are documented at the end of this article.

## Introduction

Globalization has transformed the outlook of ways to run business today. A sufficient number of professional accountants in the country is a must to manage the record of the business activities in a country (Ghani and Muhammad, 2019). However, many developing and developed countries find it challenging to keep a balance between the demand and supply of qualified accountants (Tiron-Tudor et al., 2019). Qualified accountants must have the appropriate accounting knowledge and strong technical and non-technical skills to work in a highly competitive and complex work environment (Wen et al., 2021).

The working of the business organization is becoming complex due to technology initiatives, growing composite business requirements from state agencies, a highly competitive business environment, and complex business operations (Rebele and Pierre, 2019). The demand and work routines of the accountants make the profession less attractive for the young generation “Y” and Millennials (Tiron-Tudor et al., 2019). The profile and prospects of youth vastly differ from the preceding generations in terms of workplace expectations and reward systems based on work-life (De Villers, 2020).

The ferocious and ever-changing competitive business landscape cannot attract highly talented youth to the business management profession (Gonzalez, 2021). It does not mean that the Millennials are not talented or attracted to challenging career options like professional accounting (Tiron-Tudor et al., 2019). The selection of career choices is based on the one perception of personal capacities required for the profession (Porter and Wolley, 2014). The career-related growth opportunities show that the progressive and promising growth options are relevantly available for the career aspirants and attract the youth to opt for the career option (Rebele and Pierre, 2019). Lastly, job security and stability are the significant interpreters that can attract the youth toward a specific career (Chua et al., 2019).

Attracting and retaining high talent is a must for the profession to work in a highly competitive, changing work environment (De Villers, 2020). The next generation of accountants needs to work in exceedingly competitive and vigilant to perform better in the highly technologically driven global workplace (Coe, 2016). Social and non-technical skills are also vital in empowering accounting students to pursue a career as a professional (Douglas and Gammie, 2019). The social influence and perception of control facilitate the career selection for young accounting students (Dalci and Özyapici, 2018).

### Malaysian Context

A sufficient number of qualified accountants must equal the number of new businesses registered in a country. Malaysia is a developing economy and making progress in the right direction and becoming a promising developing country, becoming developed under the Malaysian Government initiative of Economic Transformation Program (ETP) (Lee, 2018). Under the ETP initiative attracting the foreign market players to enter the Malaysian market to reap the growing market opportunities (Ghani and Muhammad, 2019). However, the growth of the local and foreign businesses in Malaysia requires a fair number of qualified accountants to manage the business activities according to the stakeholder’s needs (Lee, 2018).

Malaysian businesses as the active member of the Association of Southeast Asian Nations Economic Community (AEC) in 2015. In conjunction with the ETP, the Malaysian Government aims to become a developed country that offers reliable accounting services (Lee, 2018). An accountant refers to the stakeholders’ value in demand to ascertain that they are indeed competent and knowledgeable (Nguyen et al., 2021). Every business needs chartered accountants to sustain a prosperous financial statement, an integral business resource component (Owusu et al., 2018). One indicator of economic growth is the number of qualified accountants in the country (Jui and Wong, 2013).

The Accountants Act (1967) regulates the practice of accounting in Malaysia. It stipulates that, in order to be a qualified accountant, one must obtain more than 3 years of practical accounting working experience with a recognized training organization (Accountants Act, 1967). Therefore, a general accounting degree qualification does not make one a professional accountant until one passes the professional examinations. A total of 11 international accounting bodies are recognized in Malaysia: (1) Malaysian Institute of Certified Public Accountants (MICPA); (2) Institute of Chartered Accountants of Scotland; (3) Institute of Chartered Accountants in England and Wales; (4) Chartered Accountants Ireland; (5) Association of Chartered Certified Accountants (ACCA-United Kingdom); (6) Institute of Chartered Accountants Australia; (7) Australian Society of Certified Practising Accountants (Certified Practising Accountant Australia); (8) New Zealand Institute of Chartered Accountants; (9) Canadian Institute of Chartered Accountants; (10) Institute of Chartered Accountants of India; (11) Chartered Institute of Management Accountants (United Kingdom). The Malaysian Institute of Accountants (MIA) approves MICPA. A member of the institute is acknowledged as a “chartered accountant”, whereby “CA(M)” is used after one’s name. As a member-based organization, MIA prioritizes membership services in its day-to-day activities. The members are informed about the latest laws, practices, and guidelines that affect their profession and technical information.

Malaysia recorded approximately 33,000 new chartered accountants yearly (Lee, 2018). As reported by The Edge Malaysia, since the figure was alarming, the Performance Management and Delivery Unit (PEMANDU) had set a goal for the MIA to produce 60,000 accounting professionals by 2020 to meet the market demand (Tay, 2017). Due to the escalating number of corporations, changes in economic conditions, and increased shareholder wealth maximization, the demand for chartered accountants is rising (Coe, 2016). Therefore, MIA has collaborated with relevant stakeholders to ensure a sufficient supply of professional accountants in Malaysia.

All effort undertaken by the relevant stakeholders, only 46,000/year young professional accountant added in Malaysia. More attractiveness needs to attract young talented youths to the accounting profession (MIA, 2021). In taking the professional decision process, one may assess the perceived job characteristics, such as workload that reflects a poor working lifestyle. Besides, the current hostile environment might affect the application for Certified Professional Accountancy Qualification (CPAQ) due to the candidates’ negative perception that an accountant must work during weekends and overtime regularly (Aziz et al., 2017). Sugahara and Boland (2006) found that lower economic benefits discourage applicants from pursuing professional papers. Nonetheless, there is typically a high degree of job security with a government-approved license. The opposing views toward the profession align with the perception of choice not to adopt accounting as a career path, including adverse impressions of hectic lifestyles and prospects for finance and employment (Chua et al., 2019). Therefore, it is necessary to explore the factors influencing the intention to pursue CPAQ among the students.

The Malaysian chapter of ACCA depicts that young people are not interested in joining the accounting profession due to low career opportunities, lack of career progression, financial rewards, and low working conditions (MIA, 2021). The president delivered similar thoughts of MICAP that the young Millennials were not attracted to join the accounting profession due to long working hours and lack of workplace creativity (Lee, 2018). The scarcity of professional accountants stems from the lack of interest shown by the students and insufficient knowledge of the importance of the accounting profession among the accounting students (Ng et al., 2017). Students might demonstrate a stronger intention to become chartered accountants if they were equipped with adequate information about professional accounting at the early stage of their education (Samsuri et al., 2016). Focusing on the personal attributes and availability of accurate information to the accounting students, the current study aimed to evaluate the formation of the intention to pursue CPAQ with respect to the extended theory of planned behavior (TPB).

The subsequent section of the paper discusses the relevant literature and the development of hypotheses in the current study. Following that, the adopted method in this study is summarized in the next section. The analysis and results of the study were subsequently reported. The following section presents the discussion and conclusion of the study.

## Literature Review

The section describes the TPB and the associated factors that influence the development of the intention to pursue CPAQ. Following that, the development of hypotheses based on the literature review is discussed.

### Theory of Planned Behavior

Icek Ajzen developed the Theory of Reason Action (TRA) in 1985, and the TPB was initiated as an extension of the TRA (Ajzen, 1991). The TPB upholds those motives guide actions, but not all actions are performed, while others get adapted to various circumstances (Myburgh, 2005). Intention denotes the effort people are prepared to make, the effort they plan to accomplish, and the obstacle they may face (Ajzen, 1991). The research listed numerous issues, such as personal vulnerabilities and external factors, which may stop a person from executing the intended behavior (Ajzen, 1991). Ajzen and Madden (1986) listed some significant elements, namely attitude, perceived behavioral control, and subjective norm, to accurately predict a theoretical framework that explains how intention leads to intended behavior. Perception of behavioral control poses significant variation in the actual behavior. Attitude depicts a specific behavior or object’s positive or negative evaluation (Ajzen, 1991). Subjective norm describes the influence of peers and family members on what is considered essential, which facilitates the individual’s execution of a particular behavior (Ajzen and Madden, 1986). Perceived behavioral control represents the factors that restrict or facilitate the execution of a particular behavior (Ajzen, 1991).

The TPB has been widely employed worldwide for a range of studies (To et al., 2014). The theory has substantially impacted studies related to intentions that form human behavior (Ajzen, 1991). Therefore, the TPB appears to be the most suitable theory for this study. It ideally fits the objective to examine the significant factors in the intention of obtaining qualified certification in professional accounting.

### Determinants of Intention to Pursue Certified Professional Accountancy Qualification

The term “intention” signifies something that one plans or intends to do. An intention is pursued when a person wishes to obtain or attain a professional qualification. Due to its role as a dependent variable in the TPB, the intention is vital in assessing an actor’s performance. In simpler terms, the greater the motivation (intention) for someone to do something, the more likely the person will perform. Ajzen (1991) claimed that intention and behavioral regulation are two significant elements required to display specific behavior. The measured variables in this study were based on the existing theory and the conceptual framework. For instance, the capabilities of an accounting student would significantly affect his or her intention to pursue CPAQ. Therefore, the study had assessed variables that could significantly influence the accounting students to pursue CPAQ.

#### Capabilities

Stephenson and Yorke (1998) described capability as one’s ability to carry out intelligent personal judgment, professional ethics, self-assurance, and competence. Nonetheless, a high level of intelligence does not necessarily reflect efficient knowledge (Hammour, 2018). Capability merely determines the required level of ability to execute an activity effectively (Stephenson and Yorke, 1998). Wen et al. (2015) analyzed capacity as a factor that affected Chinese accounting students’ intention to embrace the public accounting profession.

The professional framework reveals the students’ capability to be theoretically qualified and portrays high levels of interpersonal and social psychological knowledge (Samsuri et al., 2016). Students have a dynamic way of thinking, the ability to “see” what is happening in every new situation, and the ability to recognize and assess the consequences of alternative courses of action (Yao and Hong, 2015). Every individual has varying perceptions about successfully pursuing a professional program (Gonzalez, 2021). Empirical studies have noted students’ capability as the most substantial factor influencing their intention to pursue CPAQ (Schoenfeld et al., 2017). Studies have also revealed the demand for high levels of capabilities to attain professional success, which is vital for successful professional practice in accountancy (Coe, 2016). Based on the theory and the literature, the following is proposed:

**Hypothesis 1 (H1):** Capabilities positively affect the intention to pursue CPAQ.

#### Career Opportunities

Career advancement opportunities impact the decision of students to become qualified accountants. Accounting students generally claim that the title “professional accountant” would offer them excellent work opportunities (Samsuri et al., 2016). Their perceived future employment opportunities in the accounting field can affect their accounting career choice (Uyar et al., 2011). The probability of course uptake enhances as the students perceive that a given area of the course offers exceptional work prospects (To et al., 2014).

A chartered accountant works in an organization that offers room for development, is mentally rewarding, enabling professional learning and satisfying opportunities (Samsuri et al., 2016). For instance, a starting career at a big corporation is a good start to maintain personal strength in the profession. In fact, 2 or 3 years of work experience in the big four enables one to secure the position from junior to senior (Uyar et al., 2011). The greater the expertise and knowledge one can contribute to a company, the more important one earns the status as an employee (De Villers, 2020). Large corporate firms adopt business models that promote performance and career advancements to high performers (Ghani and Muhammad, 2019).

Accounting professionals have a better opportunity for advancement when compared with the other industries (Ahmad et al., 2015). The opportunities allow them to prove that they can execute tasks efficiently (Chua et al., 2019). The provision of job opportunities is ranked at the top among Malaysian students, including accounting students (Lee, 2018). Therefore, taking the lead from the literature, career opportunity is the most effective way to influence the students to pursue CPAQ to increase the number of future professional accountants (Byrne et al., 2012). Thus, the following is hypothesized:

**Hypothesis 2 (H2):** Career opportunity positively affects the intention to pursue CPAQ.

#### Job Security and Stability

Job security is the understanding of stability and continuance of work-related issues (Nguyen et al., 2021). Work-related issues (e.g., job role, salary, and career growth) are elements that may affect the general level of job satisfaction experienced by the employee (Mueller and Kim, 2008; Myburgh, 2005). Financial stability is an integral aspect of job security as a chartered accountant (Archer, 2012). One would perceive income rise and stability upon joining a professional accounting company (Zyl and Villiers, 2011). Although accountancy offers career security and financial incentives, professional qualification suggests better salaries and more benefits to a chartered accountant (De Villers, 2020). In brief, one may enjoy handsome earnings with higher qualifications.

The three characteristics that constitute job stability are sufficient social life, working hours, and good work conditions (Schoenfeld et al., 2017). In this sense, people may expect to obtain the most significant degree of happiness in their professional lives by carrying out their professions through circumstances that fulfill as many of their goals as possible (Hammour, 2018). One will best use the interests and capacities (Ahmad et al., 2015). The findings indicate that the intention of students to pursue CPAQ demands job stability and security, which are essential for job satisfaction (Schoenfeld et al., 2017). Hence, the following hypothesis is proposed:

**Hypothesis 3 (H3):** Job security and stability positively affect the intention to pursue CPAQ.

### Attitude Toward Certified Professional Accountancy Qualification

Wen et al. (2015) defined attitude as the good or bad impression of a person toward the behavior and the belief of a party toward the behavior and the desirability to have it. Yao and Hong (2015) asserted that students’ attitudes and mindsets would change from time to time as they face different situations. Students who aim to become chartered accountants would display a better attitude toward pursuing CPAQ than those who are content in becoming a typical accountant with an undergraduate certificate of the accounting course. McDowall and Jackling (2010) depicted that when accounting students initiate the accounting course, they will exhibit a positive attitude toward accounting.

Multiple factors may affect one to pursue the CPAQ, such as intrinsic factors (e.g., interest in accounting) and extrinsic factors (e.g., advice from an expert) (Porter and Wolley, 2014). Owusu et al. (2018) claimed that accounting’s attitude primarily would be based on several significant factors, including high salary and better employment opportunities. As pursuing CPAQ ascertains one numerous benefits, he/she would have a stronger intention to pursue the qualification papers (Yusoff et al., 2011). The CPAQ syllabi should revolve around the present situation for the students to learn from case studies. Exposure to such syllabi ensures that the student can become familiar with the problem they might face in their future working lives. McDowall and Jackling (2010) postulated that the attitude of accounting students was positively linked with pursuing CPAQ. As such, the following hypothesis is formulated:

**Hypothesis 4 (H4):** Attitude toward CPAQ positively affects the intention to pursue CPAQ.

### Subjective Norm

Personal influence refers to one’s attitude toward his/her behavior, wherein social influence is formed due to the pressure from the surrounding people toward the behavior (Hammour, 2018; Owusu et al., 2018). It means that the students’ decisions may be affected by social influences, such as parents, classmates, friends, and teachers (Porter and Wolley, 2014). Students tend to rely on the referents’ opinions, which is vital to the students. Wen et al. (2015) emphasized that the students tend to satisfy demands made by their family instead of meeting their own satisfaction on their career pathway. Ahmad et al. (2015) added that students from the Asian region preferred adhering to their family opinions as they treated opinions from others with a higher value than their own opinion. To et al. (2014) postulated that most parents had already arranged the students’ educational pathway as they wanted to ensure that their children could enjoy a brighter and better future than them. The experts should influence the students’ perception toward pursuing CPAQ to deepen their knowledge about accounting and increase their skills (Nguyen et al., 2021). The students also need to acquire the knowledge of an accountant’s realistic work to intensify their intention to pursue CPAQ and become chartered accountants (Zyl and Villiers, 2011). Owusu et al. (2018) reported a substantial and significant impact on the link between subjective norm and intention to pursue CPAQ. Hence, this study considered the subjective norm vital in influencing Malaysian accounting students to pursue CPAQ. As such, the following is proposed:

**Hypothesis 5 (H5):** Subjective norm positively affects the intention to pursue CPAQ.

### Perceived Behavioral Control

Hammour (2018) defined perceived behavioral control as the confidence of a party toward his/her competency to execute the behavior or achieve the goals. Students have different recognition toward their ability, as some believe that they have adequate ability to pursue CPAQ while some have no confidence in their ability to pursue CPAQ (Myburgh, 2005). Students who aim to join the Big Four companies should be more confident about their ability and knowledge than those who do not have such a goal. Hence, those who wish to join the Big Four companies should be motivated to pursue CPAQ to secure a higher job position in the future. Perceived behavioral control is determined by prior experience and forecasted hardship to execute the target behavior (To et al., 2014).

**Hypothesis 6 (H6):** Perceived behavioral control positively affects the intention to pursue CPAQ.

## Research Methodology

This section describes the method adopted for the current research work. Adopting a cross-sectional design, a quantitative approach was explicitly considered for this study, and the proposed hypotheses were empirically tested (Baltar and Brunet, 2012). This section also discusses calculating sample size and samples from the target population. Following that, the survey development is reported in detail. The current study addressed issues of common method variance (CMV) and multivariate normality. At the end of this section, the primary analysis method is reported.

### Research Design and Sample Size

The current study had adopted the quantitative approach and the cross-sectional research design to assess the hypothesized relationships. The sample was defined based on the presumption that university students possess fundamental accounting knowledge and would like to pursue CPAQ (Liu, 2012). The G power analysis runs to determine the sample size. Based on the result of G*power version 3.1, the statistical power of 0.95 (>0.8), the effect size of 0.15, and seven model predictors showed that 153 was the ideal sample size for this study. The partial least square structural equation modeling (PLS-SEM) technique was adopted to test the hypotheses’ validity and reliability. The PLS-SEM was selected because this study had focused on measuring a theoretical framework from the prediction perspective. According to Richter et al. (2016), the “ten times rule of thumb” has been vastly applied to estimate the PLS path model by verifying the minimum requirement of the sample size. However, this rule disregards matters related to statistical power. Therefore, the required sample size for this study should exceed 300.

Data were gathered in Malaysia using the online platform due to mobility restrictions stemming from the Coronavirus Disease 2019 (COVID-19) pandemic. Hence, this study did not specify any particular location or institution as the sample venue. The study population included students aged 18–45 pursuing a Bachelor of Accounting or Accounting and Finance across all private and public universities in Malaysia. Baltar and Brunet (2012) prescribed the convenience sampling method under the non-probability approach was employed in this study. In order to ensure that the data can represent the whole population and verify data reliability, a range of samples may be selected, and the survey process may be duplicated. The convenience sampling method is also cost-efficient and saves time for data collection. The online survey was conducted by adapting the previous studies. The survey’s Google form link was shared among the target respondents from different universities using various social media platforms (Facebook and WhatsApp) and Facebook groups (university students). A convenience sampling strategy was employed, mainly because the data was collected during the COVID-19 lockdown. A total of 339 valid and completed questionnaires were retrieved and utilized for analysis.

### Survey Instrument

The online survey questionnaire (as presented in Table 1) disseminated *via* Google Forms incorporated both dependent and independent variables. The independent variables comprise attitude toward accounting, subjective norm, perceived behavioral control, career opportunities, job security, and stability. Their impact on the intention of Malaysia’s accounting students to pursue CPAQ was assessed in this study. The first section of the online questionnaire captured the respondents’ demographic information, such as gender, age, ethnicity, marital status, study year, and household income. The second part of the online questionnaire embedded independent variables. A five-point Likert scale (strongly disagree, disagree, neutral, agree, and strongly agree) was used in this study to identify the satisfaction or agreement level among respondents toward the independent and dependent variables. Additionally, a seven-point Likert scale (strongly disagree, somewhat disagree, disagree, neutral, somewhat agree, agree, and strongly agree) was applied to estimate the intention of Malaysia’s accounting students to pursue CPAQ. Different Likert point scales in this study aimed to minimize the impact of c CMV, considering that respondents concentrate and answer the survey questions differently (Podsakoff et al., 2003).

**TABLE 1:** Research instrument.

Code	Questions	Sources
CO – Item 1	I believe that being a professional accountant has higher career opportunities than a normal accountant.	Sugahara and Boland, 2006; Aziz et al., 2017; Ng et al., 2017
CO – Item 2	I am interested in being an accountant that has professional qualification as the demand of accountants is high.	
CO – Item 3	I believe that there is a high job availability for a professional accountant, thus, I have chosen my degree course as accounting.	
CO – Item 4	I think being a professional accountant can have a proper training that related to my field or industry compared to other industries.	
CA – Item 1	I believe I have the basic knowledge to pursue the Certified Professional Accountancy Qualification.	Ng et al., 2017; Hammour, 2018; Owusu et al., 2018
CA – Item 2	I have the experience that relating to accounting knowledge can help me to pursue Certified Professional Accountancy Qualification.	
CA – Item 3	I am interested to be an accountant that acquire the Certified Professional Accountancy Qualification.	
CA – Item 4	I am confident that I can pursue the Certified Professional Accountancy Qualification and become a professional accountant.	
JS – Item 1	I think being a professional accountant can earn a high salary.	Sugahara and Boland, 2006; Aziz et al., 2017; Owusu et al., 2018
JS – Item 2	I think being a professional accountant can have high job security.	
JS – Item 3	I think bringing an accounting degree holder is far not enough to secure the job as an accountant or a professional accountant.	
JS – Item 4	I believe that other fields or industries will not be as stable as the accounting field, especially for a professional accountant.	
AT – Item 1	I like to be an accountant that has pursued the Certified Professional Accountancy Qualification.	McDowall and Jackling, 2010
AT – Item 2	If I pursue the Certified Professional Accountancy Qualification, my friends would think I made a great decision for my career.	
AT – Item 3	I think accounting involves many fixed rules and regulations to comply with.	
AT – Item 4	I believe that becoming a professional accountant can acquire a lot of prestige.	
SN – Item 1	I think my peer’s influence gives a significant impact toward my career decision to pursue Certified Professional Accountancy Qualification.	Zyl and Villiers, 2011; To et al., 2014; Hammour, 2018; Owusu et al., 2018
SN – Item 2	I feel under social pressure to pursue the Certified Professional Accountancy Qualification to be a professional accountant.	
SN – Item 3	I have a closer family member/s that acquire a degree in accounting and work/s in a professional accounting organization/s.	
SN – Item 4	I want to let my family know that I am meeting their expectations by pursuing the Certified Professional Accountancy Qualification.	
PB – Item 1	I would like to pursue the Certified Professional Accountancy Qualification if the professional bodies can provide support to me.	Liu, 2012; To et al., 2014; Aziz et al., 2017; Ng et al., 2017
PB – Item 2	I believe that the difference of subculture will not become an issue for me to pursue the Certified Professional Accountancy Qualification.	
PB – Item 3	I believe that my language ability will not be an issue for me to pursue the Certified Professional Accountancy Qualification.	
PB – Item 4	I believe that the joint study program between my university and the respective professional body has given me personal satisfaction.	
INT – Item 1	I am planning to continue to complete the professional qualification paper.	Yao and Hong, 2015; Ahmad et al., 2015; Ng et al., 2017; Dalci and Özyapici, 2018
INT – Item 2	I intend to widen my knowledge about accounting.	
INT – Item 3	I intend to do more literature search or read more case studies that relate to Certified Professional Qualification knowledge.	
INT – Item 4	I would become personally satisfied if I become a professional accountant.	

*CO, career opportunities; CA, capabilities; JS, job security and stability; AT, attitudes toward accounting; SN, subjective norm; PB, perceived behavioral control; INT, intention to pursue Certified Professional Accountancy Qualification.*

### Common Method Variance

Common method variance is typical in the research domain of social science and primarily generated due to a single data collection source (Podsakoff et al., 2003). The CMV issue significantly affects the analysis results; therefore, it is recommended to evaluate this issue before conducting any main data analysis (Podsakoff et al., 2003). For this study, Harman (1976) one-factor test was conducted to address the CMV issue (Podsakoff et al., 2003). The results indicated no critical issue of CMV, as the main factor accounted for 38.65% of the total variance, below the recommended limit of 50% (Podsakoff et al., 2003).

### Multivariate Normality

The testing of multivariate normality suggests the right selection of analytical strategies. The dataset that exhibits multivariate normality can be tested using variance or co-variance-based statistical analytical tools (Cain et al., 2017). However, only a variance-based statistical tool can analyze data if the dataset does not exhibit multivariate normality (Hair et al., 2013). Following the suggestion proposed by Peng and Lai (2012), the testing of multivariate normality in the current study involved an online web power tool^1^ that verifies data normality. The results confirmed a non-normal dataset, as Mardia’s multivariate coefficient *p*-value was less than 0.05 (Cain et al., 2017).

### Data Analysis Method

Partial least squares structural equation modeling, using SmartPLS 3.1, was considered for this study to examine the gathered data. PLS-SEM is a multivariate analysis instrument that assesses path models with latent constructs (Hair et al., 2013). PLS-SEM is appropriate for non-normal and small datasets. Furthermore, unlike the covariance-based SEM, PLS-SEM can work with complex models with composites without the assumption of goodness-of-fit estimation due to its causal-predictive nature (Richter et al., 2016). For the current study, a two-step technique was considered for data analysis. The first step involves testing the reliability and validity of model constructs (Hair et al., 2013) was performed. The second step involved assessing the structural model, and testing hypotheses (Richter et al., 2016). The model estimations were performed based on the results of *r*^2^, *Q*^2^, and effect size (*f*^2^), which describe the path effects of exogenous constructs to endogenous construct (Hair et al., 2013).

## Findings

This section reports the obtained analysis results, including the demographic profile of the respondents in this study. Following that, the section describes the reliability and validity of the model constructs. The appropriateness of the reliability and validity of the model constructs were first determined before the study proceeded to the testing of hypotheses *via* path analysis. The results of multiple group analysis are reported at the end of this section.

### Demographic Profile of Respondents

Table 2 presents the demographic profile of the respondents (a sample of 339 Malaysian accounting students). The majority of the respondents were female (58.1%). In addition, most of the respondents were of the age group of 21–25 years old (57.8%). About 26.0% of the total respondents were below 21 years old. Meanwhile, 8.8 and 4.1% of the total respondents were between 26 and 30 years old and 31 and 35 years old, respectively. Besides that, 65.5% of the total respondents were of Chinese ethnicity, followed by Malay (13.6%) and Indian (12.1%) ethnicity groups. The remaining respondents (8.8%) were of other ethnic groups. Next, a large proportion of the respondents were single (92.3%). More than 50% of the respondents were in their third year of study, and only 12.7% of the total respondents were first-year students. Most of the respondents reported a household income below RM 5,000 (50.4%), followed by those with a household income of between RM 5,000 and RM 10,000 (34.2%).

**TABLE 2:** Demographic profile of respondents.

	*N*	%		*N*	%
**Gender**			**Marital status**		
Female	197	58.1	Single	313	92.3
Male	142	41.9	Married	25	7.4
Total	339	100.0	Divorced	1	0.3
**Age group**			Total	339	100.0
Below 21 years	88	26.0	**Year of study**		
21–25 years	196	57.8	Year 1	43	12.7
26–30 years	30	8.8	Year 2	52	15.3
31–35 years	14	4.1	Year 3	176	51.9
36–40 years	7	2.1	Year 4	68	20.1
>40 years	4	1.2	Total	339	100.0
Total	339	100.0	**Average monthly household income**		
**Ethnicity**			Below RM 5,000	171	50.4
Malay	46	13.6	RM 5,001–RM 10,000	116	34.2
Chinese	222	65.5	RM 10,001–RM 15,000	21	6.2
Indian	41	12.1	RM 15,001–RM 20,000	14	4.1
Others	30	8.8	RM 20,001–RM 25,000	14	4.1
Total	339	100.0	More than RM 25,000	3	0.9
			Total	339	100.0

### Reliability and Validity

Table 3 shows the reliability of the items used in this study upon implementing the descriptive statistics and the criteria used. The mean and standard deviation for all the variables are tabulated (capabilities, career opportunities, job security and stability, attitude toward CPAQ, subjective norm, perceived behavioral control, and intention to pursue CPAQ). Although the mean values for capabilities and subjective norms were relatively low, their standard deviation outcomes were high. It signified that not all the accounting students had similar knowledge regarding their capabilities and subjective norms, which could influence their intention to pursue CPAQ.

**TABLE 3:** Reliability and validity.

Variables	No. items	Mean	SD	CA	DG *rho*	CR	AVE
CAP	4	3.995	1.078	0.937	0.939	0.955	0.841
COP	4	4.252	0.898	0.900	0.904	0.930	0.769
JSS	4	4.309	0.792	0.887	0.892	0.922	0.747
ATT	4	4.261	0.823	0.877	0.901	0.915	0.729
SUN	4	3.570	0.875	0.724	0.866	0.821	0.582
PBC	4	4.260	0.874	0.928	0.930	0.949	0.823
ICPAQ	4	5.566	1.443	0.937	0.939	0.955	0.841

*CAP, capabilities; COP, career opportunities; JSS, job security and stability; ATT, attitudes toward Certified Professional Accountancy Qualification; SUN, subjective norm; PBC, perceived behavioral control; ICPAQ, intention to pursue a Certified Professional Accountancy Qualification; SD, standard deviation; CA, Cronbach’s alpha; DG rho, Dillon-Goldstein’s rho; CR, composite reliability; AVE, average variance extracted.*

*Source: Author’s data analysis.*

Generally, Cronbach’s alpha is a measurement that determines the internal consistency reliability and the relationship among datasets. Based on the Cronbach’s alpha reliability analysis, all variables scored more than 0.7, indicating that all the variables were acceptable and reliable. This measurement was used for multipoint-scaled items inclusive of the Likert scale. The other measurement used in this study to determine internal consistency reliability is composite reliability (CR). When CR values are equal to or more than 0.7, they indicate that the model has sufficient reliability (Hair et al., 2013). Table 3 shows that the CR and Dillon-Goldstein *rho* values for all variables had exceeded 0.8 and 0.7, respectively, signifying acceptable reliability for all the variables.

The AVE is an essential tool that determines convergent validity. Values of AVE should be equal to or greater than 0.5 to indicate acceptance, which means that the amount of variance captured from the latent variable is at least 50% or above. As shown in Table 3, the AVE values for all variables exceeded 0.5. Next, the values of loading and cross-loading, as portrayed in Table 4, were above 0.7, depicting reliable data. The cross-loadings indicate that all loadings of indicators are higher than the total cross-loadings, verifying discriminant validity. Discriminant validity is typically used to assess if a study construct has no relationship or is unrelated. Discriminant validity is determined only after successfully establishing CR, Cronbach’s alpha, and AVE.

**TABLE 4:** Loadings and cross-loading.

Code	CAP	COP	JSS	ATT	SUN	PBC	ICAPQ
CAP – Item 1	*0.925*	0.721	0.588	0.676	0.547	0.689	0.783
CAP – Item 2	*0.886*	0.634	0.491	0.572	0.484	0.604	0.750
CAP – Item 3	*0.929*	0.818	0.659	0.782	0.595	0.769	0.841
CAP – Item 4	*0.928*	0.748	0.645	0.743	0.569	0.738	0.832
COP – Item 1	0.654	*0.859*	0.699	0.699	0.505	0.688	0.670
COP – Item 2	0.751	*0.907*	0.657	0.728	0.570	0.724	0.766
COP – Item 3	0.716	*0.880*	0.653	0.729	0.553	0.714	0.686
COP – Item 4	0.679	*0.862*	0.797	0.810	0.600	0.751	0.674
JSS – Item 1	0.523	0.649	*0.859*	0.723	0.457	0.654	0.541
JSS – Item 2	0.511	0.687	*0.870*	0.739	0.454	0.612	0.526
JSS – Item 3	0.606	0.711	*0.891*	0.784	0.579	0.745	0.633
JSS – Item 4	0.602	0.703	*0.837*	0.734	0.589	0.639	0.614
ATT – Item 1	0.809	0.819	0.745	*0.885*	0.658	0.829	0.778
ATT – Item 2	0.714	0.746	0.801	*0.902*	0.658	0.782	0.704
ATT – Item 3	0.458	0.630	0.665	*0.789*	0.503	0.611	0.499
ATT – Item 4	0.539	0.660	0.733	*0.835*	0.561	0.667	0.587
SUN – Item 1	0.527	0.552	0.524	0.613	0.904	0.567	0.545
SUN – Item 2	0.419	0.462	0.456	0.510	0.852	0.450	0.454
SUN – Item 3	−0.032	−0.098	−0.090	−0.084	0.646	−0.097	0.002
SUN – Item 4	0.605	0.632	0.598	0.703	0.873	0.689	0.615
PBC – Item 1	0.735	0.780	0.729	0.820	0.670	*0.911*	0.790
PBC – Item 2	0.652	0.711	0.662	0.739	0.591	*0.911*	0.721
PBC – Item 3	0.643	0.678	0.682	0.735	0.534	*0.907*	0.702
PBC – Item 4	0.739	0.794	0.716	0.807	0.596	*0.898*	0.773
ICAPQ – Item 1	0.855	0.779	0.646	0.741	0.603	0.794	*0.924*
ICAPQ – Item 2	0.758	0.673	0.585	0.639	0.504	0.711	*0.901*
ICAPQ – Item 3	0.780	0.692	0.593	0.646	0.524	0.692	*0.927*
ICAPQ – Item 4	0.811	0.777	0.642	0.779	0.642	0.819	*0.916*

*CAP, capabilities; COP, career opportunities; JSS, job security and stability; ATT, attitudes toward Certified Professional Accountancy Qualification; SUN, subjective norm; PBC, perceived behavioral control; ICPAQ, intention to pursue a Certified Professional Accountancy Qualification.*

*The italic values in the matrix above are the item loadings and others are cross-loadings.*

*Source: Author’s data analysis.*

### Path Analysis

The *r*^2^ value, which represents the degree of explained variance, was 0.827, which indicated that the six factors (i.e., CAP, COP, JSS, ATT, SUN, and PBC) could explain a significant proportion (82.7%) of the variation for students’ intention to pursue CPAQ.

As depicted in Table 5, the path between the CAP and ICPAQ (beta = 0551, *t*-value = 10.336, *p* = 0.000) depicts CAP’s positive and significant impact on the ICPAQ statistical support to accept the H1. The *f*^2^ value of 0.546 revealed a higher effect size. Following, the path between the COP and ICPAQ (beta = 0.105, *t*-value = 1.828, *p* = 0.034) confirms that career opportunities significantly and positively impact the intention to pursue the CPAQ. The outcome suggests admitting the H2. The *f*^2^ value of 0.013 displayed a small effect size. Subsequently, the path between the JSS and ICPAQ (beta = −0.020, *t*-value = 0.330, *p* = 0.371) approves that job security and stability negatively and insignificantly influence the intention to pursue the CPAQ. The consequence advocates not to accept the H3. The *f*^2^ value of 0.001 displayed a small effect size. Next, the path between the ATT and ICPAQ (beta = −0.049, *t*-value = 0.663, *p* = 0.254) approves that attitude toward ICPAQ insignificantly and negatively influences the intention to pursue the CPAQ. The result proposes not to accept the H4. The *f*^2^ value of 0.002 displayed a weak effect size. Subsequent, the path between the SUN and ICPAQ (beta = 0.043, *t*-value = 1.207, *p* = 0.114) confirms that subjective norms insignificantly but positively impact the intention to pursue the CPAQ. The outcome offers not to accept the H5. The *f*^2^ value of 0.005 displayed a weak effect size. Lastly, the path between the PBC and ICPAQ (beta = 0.347, *t*-value = 6.039, *p* = 0.000) settles that the perceived behavioral control significantly and positively affects the intention to pursue the CPAQ. The outcome proposes to admit the H6. The *f*^2^ value of 0.152 exhibited a moderate effect size. Table 5 presents the results of path coefficients.

**TABLE 5:** Path coefficients.

Hypothesis		Beta	CI – Min	CI – Max	*T*	*P*	*r* ^2^	*f* ^2^	Decision
H_1_	CAP → ICPAQ	0.551	0.456	0.631	10.336	0.000		0.546	Accept
H_2_	COP → ICPAQ	0.105	0.015	0.203	1.828	0.034		0.013	Accept
H_3_	JSS → ICPAQ	−0.020	−0.122	0.079	0.330	0.371	0.827	0.001	Reject
H_4_	ATT → ICPAQ	−0.049	−0.163	0.082	0.663	0.254		0.002	Reject
H_5_	SUN → ICPAQ	0.043	−0.012	0.106	1.207	0.114		0.005	Reject
H_6_	PBC → ICPAQ	0.347	0.240	0.429	6.039	0.000		0.152	Accept

*CAP, capabilities; COP, career opportunities; JSS, job security and stability; ATT, attitudes toward Certified Professional Accountancy Qualification; SUN, subjective norm; PBC: perceived behavioral control; ICPAQ, intention to pursue a Certified Professional Accountancy Qualification.*

*Source: Author’s data analysis.*

### Multi-Group Analysis

Multiple group analysis was conducted to compare the outcomes for various groups based on gender and years of study in accounting. The multi-group analysis enables one to assess the model’s critical paths differences based on the respondents’ gender and study year. Table 6 presents the path values for groups of two with variations within gender and study year, along with *p*-values, as Henseler et al. (2009) prescribed. The PLS-based method to multi-group analysis (PMGA) was strongly required to underpin the outcomes, which appeared essential for this study to compare the model’s parameters across gender and study year (Henseler et al., 2009). Besides, the student’s gender and year of enrollment in the accounting program did not affect the study path.

**TABLE 6:** Multi-group analysis.

	Female		Male		Difference		
	Beta	*p-*Value	Beta	*p-*Value	Beta	*p-*Value	Decision
CAP → ICAPQ	0.538	0.000	0.591	0.000	−0.053	0.290	No difference
COP → ICAPQ	0.067	0.211	0.165	0.040	−0.099	0.217	No difference
JSS → ICAPQ	−0.067	0.192	0.062	0.228	−0.130	0.124	No difference
ATT → ICAPQ	0.016	0.436	−0.180	0.065	0.196	0.096	No difference
SUN → ICAPQ	0.043	0.191	0.050	0.207	−0.007	0.453	No difference
PBC → ICAPQ	0.377	0.000	0.299	0.001	0.078	0.253	No difference

	**1–2 years**		**3–4 years**		**Difference**		
	**Beta**	***p-*Value**	**Beta**	***p-*Value**	**Beta**	***p-*Value**	**Decision**

CAP → ICAPQ	0.587	0.000	0.409	0.000	0.179	0.076	No difference
COP → ICAPQ	0.153	0.197	0.153	0.015	0.000	0.471	No difference
JSS → ICAPQ	−0.185	0.143	0.003	0.481	−0.188	0.144	No difference
ATT → ICAPQ	−0.206	0.144	0.056	0.236	−0.262	0.098	No difference
SUN → ICAPQ	0.129	0.099	0.009	0.389	0.120	0.118	No difference
PBC → ICAPQ	0.411	0.005	0.364	0.000	0.047	0.388	No difference

*CAP, capabilities; COP, career opportunities; JSS, job security and stability; ATT, attitudes toward Certified Professional Accountancy Qualification; SUN, subjective norm; PBC, perceived behavioral control; ICPAQ, intention to pursue a Certified Professional Accountancy Qualification.*

*Source: Author’s data analysis.*

The results of multiple group analysis suggested no significant differences across the sample groups in terms of gender and year of enrollment.

### Predictive Assessment

The model displayed high predictive power, as most of the *Q*^2^_predict_ values exceeded zero. The results of Q^2^_predict_ suggested that the naïve PLS-SEM model executed better than the LM scale, and most of the LM benchmark yielded more errors than the PLS-SEM model. Based on these results presented in Table 7, the study offered critical evidence that the proposed PLS-SEM model performs well in predicting ICPAQ.

**TABLE 7:** Results of predictive model assessment.

	*Q* ^2^_predict_	RMSE (PLS-SEM)	RMSE (LM)	Difference	Predictive power
ICPAQ – Item 1	0.771	0.770	0.819	−0.049	High predictive power
ICPAQ – Item 2	0.602	0.926	0.960	−0.034	
ICPAQ – Item 3	0.614	0.971	1.009	−0.038	
ICPAQ – Item 4	0.742	0.843	0.830	0.013	

*ICPAQ, intention to pursue Certified Professional Accountancy Qualification; RMSE, root mean square error; PLS-SEM, partial least squares structural equation modeling; LM, linear regression model.*

*Source: Author’s data analysis.*

## Discussion

The current study empirically evaluates the effect of capabilities, career opportunities, job security and stability, attitude toward CPAQ, subjective norm, and perceived behavior control on the intention of students to pursue CPAQ in Malaysia.

The study confirms that the perception of capabilities significantly promotes the intention to pursue the CPAQ. The current result suggests accepting the H1. The study outcome accords with the results suggested by Schoenfeld et al. (2017) that perception of higher personal capabilities nurtures the inclination to engage in account qualification. Personal capacity and aptitude facilitate learning and promote the positive predispositions to engage in higher education or professional qualification (Tiron-Tudor et al., 2019).

Next, the perception of career opportunities significantly instigates the intention to build a career in professional accountancy. The current outcome offers substantial sustenance to accept H2. The current finding coincides with the result documented by Samsuri et al. (2016) that the perception of career opportunities instigates the intention to pursue the CPAQ. The better career prospect harness the motivation to pursue higher education and engage in professional qualifications to have a promising career (Ghani and Muhammad, 2019).

Job security and stability in this study exhibited insignificant, adverse effects on the intention to pursue CPAQ among Malaysian accounting students, signifying that job security and stability were projected as weak determinants for the intention to pursue CPAQ. It offers no support to accept the H3. Our study result deviates from the findings suggested by Tiron-Tudor et al. (2019) that job insecurity and instability may not influence the intention to pursue accounting qualifications among the young Millennials.

Additionally, attitude toward CPAQ in the study exhibited insignificant, negative effects on the intention to pursue CPAQ among Malaysian accounting students; the result depicts not to accept H4. Our study outcome contradicts with result posted by Owusu et al. (2018) that the positive attitude nurtures the intention to pursue professional accounting qualification. The attitude toward the CPAQ among the current study respondents was low, and accounting students were not inclined to pursue the CPAQ.

Meanwhile, subjective norms had a positive but insignificant effect on the students’ intention to pursue CPAQ, and it offers no support to accept the H5. The current finding matches the results Porter and Wolley (2014) postulated that the subject norms are not appropriately instigating intention to pursue the accounting major among the United States undergraduate students.

Lastly, the current study’s finding establishes that the perceived behavioral control significantly impacts the intention to pursue CAPQ among the study respondents. The study offers statistical support to accept H6. The current work achieves its support from the work of To et al. (2014) that the perception of support from peers and family promotes the intention to pursue the accounting qualification among the university students.

Subsequently, the multigroup analysis revealed that the respondent gender insignificantly differs among the group based on gender. The analysis suggests that the respondent’s gender offers no variance among the study respondent for influencing the intention to pursue CPAQ. Moreover, the multigroup analysis revealed that the year of enrollment also insignificantly diverges among the group based on the year of enrollment. The result suggests that the year of enrollment remains the same among the study respondents for the factors influencing the intention to pursue CPAQ.

Lastly, the predictive assessment of the model poised that the current model is suitable to perform prediction as the most of factor for ICPAQ yield lowers errors than the LM. The finding confirms that the PLS-SEM performs better than the LM. The current model is good for out-of-sample prediction representing the intention to pursue the CPAQ with the personal factors of CAP, COP, JSS, and factors taken from TPB, i.e., ATT, SUN, and PBC.

Studies concerning students’ perceptions of becoming chartered accountants have been vastly conducted on Western-based accounting structures, which generated poor results (Lee, 2018). To the best of the authors’ knowledge, this study is the first to incorporate TPB as the underlying basis to investigate the intention to pursue CPAQ within the Malaysian context. As such, this study had examined the effect of accounting students’ capabilities, career opportunities, job security and stability, attitude, subjective norm, and perceived behavioral control on their intention of pursuing CPAQ. The current outcomes substantially enrich the literature of accounting students by offering empirical evidence regarding the effects that create intention among accounting students to pursue CPAQ.

Essentially, accounting students must be equipped to continually update themselves on the latest developments in technical accounting matters and other vital areas that may be directly or indirectly relevant to their career progress. The study outcomes have implications on how academic and education reforms may be restructured to stress active student learning to develop attitudes consistent with the culture of self-directed or lifelong learning critical to succeed in the increasingly challenging work environment. The reforms should aim to develop a multiple-competence workforce as businesses seek highly adaptable employees who can look beyond the numbers to achieve congruence at the workplace and in the environment. Accounting education reforms should emphasize continuous updating and enhancing current knowledge, apart from improving the ability of graduates to apply new knowledge to real-world situations or professional practices.

## Conclusion

The demand for chartered accountants has been expected to increase due to economic changes, mushrooming organizations, and the wealth of company shareholders. Malaysia needs to have more than 60,000 professional accountants yearly to meet the expected growth in the Malaysian economy (MIA, 2021). The study had assessed several factors that influenced the intention of accounting students to pursue CPAQ. The study examined the effect of attitude toward accounting, subjective norm, and perceived behavioral control, which were retrieved from TPB, along with other factors, namely capabilities, career opportunities, and job security and stability extending the TPB to evaluate the intention of accounting students to pursue CPAQ. The study outcomes revealed that the intention of accounting students in Malaysia to pursue CPAQ was positively influenced by the factors of capabilities, career opportunities, and perceived behavioral control. Hence, the Malaysian government should encourage and support accounting students in their pursuit of CPAQ by providing fee concession, loan facilities, and stipends.

Besides that, large accounting firms may consider offering financial support to accounting students, harnessing their interest to pursue CPAQ (Tay, 2017). Accounting firms need to partner with accounting educational institutions to offer internships and hands-on experience to accounting students (Gonzalez, 2021). That helps improve the students’ perception of the accounting profession and learn first-hand knowledge from the accounting experts (Douglas and Gammie, 2019). The professional accounting bodies (MIA, ACCA, and others) must collaborate with universities and colleges by allowing the students to get exempt from their specific accounting professional papers (Ghani and Muhammad, 2019). It can help students save their time and money from taking professional papers after their degree or diploma accounting courses. Collaboratives efforts from accounting professional bodies, large accounting firms accounting educational institutions can harness the accounting students to pursue professional accounting qualifications and have the appropriate knowledge and skills to pursue a career as a professional accountant.

There were four limitations associated with the current study. Firstly, the study assessed factors that affect the intention to pursue CPAQ among accounting students in Malaysia, but the generalization of quantitative survey results was limited. Therefore, it is recommended that future research accumulate longitudinal data to highlight the effects of factors that affect the students’ intention to pursue CPAQ. Secondly, family members, peers, and faculty members may influence students’ career choices. Hence, it is recommended for future research to consider incorporating other social influences, peer pressure, and social norm factors that may contribute to the student’s intention to pursue CPAQ. Thirdly, personal inclinations significantly influence one’s intention to adopt a specific behavior. Therefore, it is suggested for future research to incorporate personal inclination factors that may influence the intention to pursue CPAQ. Future researchers may also replicate the study for other professional accounting qualifications like ACCA and CA(M).

## Data Availability Statement

The original contributions presented in the study are included in the article/Supplementary Material, further inquiries can be directed to the corresponding author.

## Ethics Statement

This study has been performed in accordance with the Declaration of Helsinki. Informed consent for participation was obtained from respondents who participated in the survey. The respondents who participated in the survey online (using google form) were asked to read the ethical statement posted on the top of the form (There is no compensation for responding, nor is there any known risk. In order to ensure that all information will remain confidential, please do not include your name. Participation is strictly voluntary, and you may refuse to participate at any time) and proceed only if they agree. No data was collected from anyone under 18 years old.

## Author Contributions

TC, TS, NH, and AS: conceptualization, methodology, data collection, and writing – original draft. AA and QY: methodology (instrument), data analysis, and writing – editing and revision. All authors contributed to the article and approved the submitted version.

## Conflict of Interest

The authors declare that the research was conducted in the absence of any commercial or financial relationships that could be construed as a potential conflict of interest.

## Publisher’s Note

All claims expressed in this article are solely those of the authors and do not necessarily represent those of their affiliated organizations, or those of the publisher, the editors and the reviewers. Any product that may be evaluated in this article, or claim that may be made by its manufacturer, is not guaranteed or endorsed by the publisher.

## Footnotes

1
https://webpower.psychstat.org/wiki/tools/index
